# Postoperative computed tomography‐derived bundle morphology ratios are associated with noncontact graft failure after double‐bundle anterior cruciate ligament reconstruction in adolescents

**DOI:** 10.1002/jeo2.70864

**Published:** 2026-07-28

**Authors:** Yuko Takeuchi, Ryosuke Kawai, Hideki Hiraiwa, Takashi Tsukahara

**Affiliations:** ^1^ Department of Orthopedic Surgery Asahi University Hospital Gifu Japan

**Keywords:** anterior cruciate ligament, double‐bundle reconstruction, graft failure, imaging morphology, risk prediction

## Abstract

**Purpose:**

To evaluate whether postoperative computed tomography (CT)‐derived bundle‐specific morphology ratios are associated with noncontact graft failure following anatomic double‐bundle anterior cruciate ligament (ACL) reconstruction in adolescents.

**Methods:**

This single‐centre retrospective cohort study included 133 consecutive patients aged 15–19 years who underwent primary anatomic double‐bundle ACL reconstruction with routine postoperative CT at 1 week postoperatively and a minimum follow‐up of 24 months; all patients had a complete 2‐year outcome assessment. Patients with a lateral femoral condyle ratio (LFCR) ≥ 62.18% were excluded, as this threshold has been associated with substantially elevated graft rerupture risk [2]. Only noncontact graft failures within 2 years were considered events. Preoperative MRI was used to assess femoral morphology, and postoperative CT was used to quantify anteromedial (AMR) and posterolateral (PLR) bundle morphology ratios. Receiver operating characteristic analysis and multivariable logistic regression were performed to evaluate discriminative ability and independent associations with graft failure.

**Results:**

Both AMR and PLR were significantly associated with graft failure and demonstrated moderate‐to‐good discriminative ability (area under the curve 0.804 and 0.799, respectively). In multivariable analysis adjusting for age, sex, BMI, activity level and meniscal injury status, only AMR remained associated with graft failure in an exploratory multivariable model (OR: 1.15 per 0.01‐unit increase, 95% CI: 1.02–1.31; *p* = 0.026). Intraobserver reliability was good to excellent across all parameters (intraclass correlation coefficients 0.878–0.949).

**Conclusion:**

Postoperative CT‐derived bundle morphology ratios are associated with noncontact graft failure following anatomic double‐bundle ACL reconstruction, with AMR bundle morphology demonstrating the strongest association in an exploratory multivariable model. These preliminary findings suggest that quantitative morphology ratios may be useful for future risk stratification, but prospective validation is required before clinical implementation.

**Level of Evidence:**

Level III, retrospective cohort study.

**Abbreviations**
ACL: anterior cruciate ligament
AM: anteromedial
AMR: anteromedial ratio
AUC: area under the curve
PL: posterolateral
PLR: posterolateral ratio
ROC: receiver operating characteristic

## INTRODUCTION

Graft failure following anterior cruciate ligament (ACL) reconstruction remains a significant clinical challenge and is influenced by patient‐specific risk factors and structural morphology [10, 11, 13]. Anatomic double‐bundle reconstruction techniques aim to more closely reproduce native knee biomechanics and improve rotational stability [1, 2, 12]; however, reinjury rates remain substantial in younger active populations [10, 11].

Recent investigations have emphasised the role of osseous morphology, including lateral femoral condyle configuration and posterior tibial slope, in predicting primary ACL injury and rerupture risk [3, 4, 5, 6, 7, 8, 9]. Nevertheless, most prior studies have focused on native bony anatomy rather than postoperative graft configuration. Bundle‐specific morphological characteristics derived from routine clinical imaging, which may more directly reflect graft loading conditions and surgical positioning, remain insufficiently investigated.

Although single‐bundle reconstruction remains the predominant technique in many Western countries, anatomic double‐bundle reconstruction has been widely adopted in Japan and parts of East Asia, supported by evidence demonstrating superior rotational stability and biomechanical fidelity to the native ACL [1, 2, 12, 13]. This institutional and regional experience provides a unique opportunity to investigate bundle‐specific postoperative morphology in a sufficiently large consecutive cohort.

The anteromedial (AMR) and posterolateral (PLR) bundle morphology ratios, as defined in this study, represent the relative anterior position of each femoral tunnel within the condylar arc. A higher ratio indicates a more anteriorly positioned tunnel, which may result in a more vertical graft orientation, altered graft tension during knee flexion and potential impingement against the intercondylar roof. These biomechanical consequences may increase cyclic loading on the graft and predispose it to failure, particularly during dynamic activities in younger patients. The purpose of this study was to evaluate whether postoperative CT‐derived AMR and PL bundle morphology ratios are associated with noncontact graft failure following anatomic double‐bundle ACL reconstruction in adolescents.

We hypothesised that bundle‐specific morphology ratios would be associated with noncontact graft failure.

## METHODS

### Study design

This was a single‐centre retrospective cohort study conducted between 2016 and 2022 and conducted in accordance with the Declaration of Helsinki and was approved by the Ethics Committee of Asahi University Hospital (Research No. 2026‐01‐01).

### Patient selection

**Figure 1:**
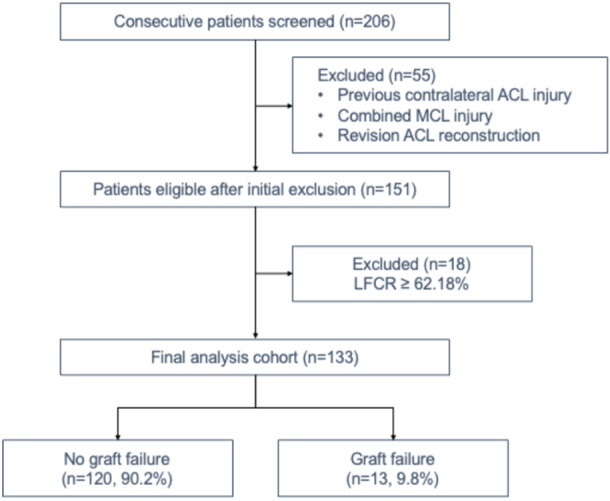
Study flow diagram. Flowchart illustrating patient screening and inclusion in the final analysis. Of 206 consecutive patients initially screened, 55 were excluded due to previous contralateral ACL injury, combined MCL injury or revision ACL reconstruction. A further 18 patients were excluded due to a lateral femoral condyle ratio (LFCR) ≥ 62.18%, resulting in 133 patients included in the final cohort. ACL, anterior cruciate ligament.

Inclusion criteria were: (1) patients aged 15–19 years who underwent primary anatomic double‐bundle ACL reconstruction, (2) graft failures resulting from a noncontact mechanism and (3) a minimum follow‐up of 24 months. Exclusion criteria were: previous contralateral ACL injury, combined MCL injury, revision ACL reconstruction and a lateral femoral condyle ratio (LFCR) ≥ 62.18%, as prior literature has associated this threshold with a substantially elevated risk of graft rerupture [3]. After applying all predefined criteria, 133 patients were included in the final analysis (Figure 1).

### Surgical procedure

All procedures were performed using an anatomic double‐bundle ACL reconstruction technique via a trans‐portal inside‐out approach by multiple experienced knee surgeons at our institution using standardised surgical protocols. Hamstring tendons (semitendinosus and gracilis) were harvested and prepared as quadrupled grafts. The anteromedial (AM) bundle was reconstructed using the semitendinosus tendon (median graft diameter 7 mm) with a femoral tunnel diameter of 7 mm, and fixed with a staple (Meira, Nagoya, Japan) at near full extension. The PL bundle was reconstructed using the gracilis tendon (median graft diameter 6 mm) with a femoral tunnel diameter of 6 mm, and fixed with a staple (Meira, Nagoya, Japan) at near full extension. Femoral tunnel positions were determined based on anatomic landmarks corresponding to the native AM and PL bundle footprints. As all surgeons followed the same standardised protocol throughout the study period, a systematic learning curve effect is considered unlikely; however, potential confounding from intersurgeon variability cannot be fully excluded and is acknowledged as a limitation.

### Outcome definition

Graft failure was defined as recurrent instability confirmed by clinical examination and imaging, or revision ACL reconstruction within 2 years postoperatively, provided that the reinjury occurred through a noncontact mechanism. Cases of graft rupture associated with direct contact trauma or traumatic events unrelated to intrinsic knee instability were excluded from the failure group. Failure events were identified through routine clinical follow‐up at our institution, including scheduled outpatient visits and chart review. Mechanism of reinjury was determined based on medical record documentation and patient‐reported injury circumstances at the time of clinical evaluation. Two authors independently reviewed each case to confirm noncontact mechanism classification; cases with ambiguous mechanism were excluded from the failure group.

## IMAGING MEASUREMENTS

### Preoperative magnetic resonance imaging (MRI) assessment

Preoperative MRI was used to quantify native femoral morphology. The LFCR was measured on standardised sagittal images according to the method described by Gao et al. Image slices were selected based on reproducible anatomical landmarks, and measurements were performed using digital imaging software under identical viewing conditions. Ratios were calculated relative to defined femoral condyle reference distances to reduce interindividual scaling effects.

### Postoperative computed tomography (CT) assessment

Routine postoperative CT was performed at 1 week after surgery in all patients using a standardised protocol (120 kVp, 150 mA, slice thickness 2.5 mm). This modality was selected because metal artifact from surgical hardware renders MRI‐based tunnel measurement unreliable in the postoperative setting. Slice selection and distal femoral shaft axis determination were performed according to the method described by Gao et al. The total condylar length (D1) was defined as the distance between the anterior and posterior cortical margins. Distances from the posterior condylar margin to the anterior edge of the AM and PL femoral tunnels were defined as D_AM_ and D_PL_, respectively. Morphology ratios were calculated as follows:

$$\text{AMR}\;={\text{D}}_{\text{AM}}/\text{D1}$$

$$\text{PLR}\;={\text{D}}_{\text{PL}}/\text{D1}$$

**Figure 2:**
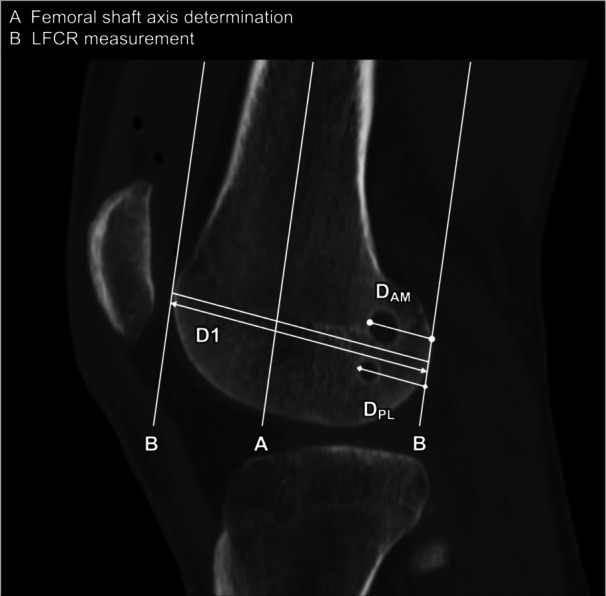
Measurement of postoperative CT‐derived bundle morphology ratios. Representative sagittal CT images illustrating the measurement of anteromedial (AM) and posterolateral (PL) bundle morphology ratios. Distances from the posterior condylar margin to the anterior edge of the AM and PL femoral tunnels (Dᴀ^M^ and Dᴘ^L^, respectively) were normalised to the total condylar length (D1) to calculate AMR (Dᴀ^M^/D1) and PLR (Dᴘ^L^/D1). The figure also illustrates the femoral shaft axis determination method (vertical lines A and B) adapted from Gao et al. [3], which was originally described for LFCR measurement but is used here solely to define the reference axis for bundle morphology ratio calculation. AMR, anteromedial ratio; LFCR, lateral femoral condyle ratio; PLR, posterolateral ratio.

Representative measurements are illustrated in Figure 2.

### Reproducibility analysis

All imaging measurements were performed by a single observer blinded to clinical outcomes and repeated after an interval of 2 weeks. Intraobserver reliability was assessed using intraclass correlation coefficients (ICC). ICC was calculated using a two‐way mixed‐effects model with absolute agreement. Agreement between repeated measurements was further evaluated using Bland–Altman analysis.

### Statistical analysis

Continuous variables were assessed for distribution and compared using the Mann–Whitney *U*‐test where appropriate. Categorical variables were compared using chi‐square or Fisher's exact tests. Receiver operating characteristic (ROC) curve analysis was performed to evaluate the discriminative ability of bundle morphology parameters for graft failure in this cohort. Area under the curve (AUC) with 95% confidence intervals was calculated using the DeLong method. Optimal cutoff values were determined using Youden's index.

Multivariable logistic regression analysis was conducted as an exploratory analysis to evaluate the associations between bundle morphology ratios and graft failure after adjustment for potential confounders. Covariates included age, sex, body mass index, activity level (Tegner score) and meniscal injury status. Results were reported as odds ratios (ORs) with 95% confidence intervals. Model discrimination was assessed using AUC, and calibration was evaluated with the Hosmer–Lemeshow goodness‐of‐fit test. Variance inflation factors were calculated to assess multicollinearity. AMR and PLR were rescaled (×100) for logistic regression analysis to express ORs per 0.01‐unit increase.

Statistical analyses were performed using EZR (Saitama Medical Centre, Jichi Medical University), a graphical interface for R software. Statistical significance was set at *p* < 0.05.

## RESULTS

### Patient characteristics

A total of 206 patients were initially screened, of whom 55 were excluded due to previous contralateral ACL injury, combined MCL injury or revision ACL reconstruction, leaving 151 eligible patients. Of these, 18 were further excluded due to LFCR ≥ 62.18%, resulting in 133 patients included in the final analysis, of whom 13 (9.8%) experienced graft failure within 2 years postoperatively (Figure 1). Baseline demographic and clinical characteristics are summarised in Table 1.

**Table 1:** Patient demographics and baseline characteristics. Demographic and clinical characteristics of the study population, including age, sex, BMI, activity level, graft type, meniscal status (all repaired) and injury outcome within 2 years. Values are presented as median (IQR) or number (%).

Variable	Nonfailure (*n* = 120)	Failure (*n* = 13)	*p*‐value
Age (years)	16 (16–17)	16 (16–19)	0.47
Sex (male)	42 (35.0)	6 (46.2)	0.55
BMI (kg/m^2^)	21.4 (20.3–23.1)	22.6 (21.2–24.8)	0.14
Side (right)	61 (50.8)	6 (46.2)	0.78
Graft type (STG)	120 (100)	13 (100)	1.00
Meniscus injury			0.57
None	53 (44.2)	7 (53.8)	
Medial	29 (24.2)	3 (23.1)	
Lateral	46 (38.3)	3 (23.1)	
Both	8 (6.7)	0 (0)	
Tegner score	9 (5–9)	7 (7–9)	0.16

*Note*: Categories are not mutually exclusive.

Abbreviations: BMI, body mass index; IQR, interquartile range; MCL, medial collateral ligament; STG, semitendinosus‐gracilis tendon.

### Morphological measurements and graft failure

Postoperative CT‐derived bundle morphology ratios were significantly greater in patients who experienced graft failure.

For the AM bundle, the median AMR was 0.1397 (IQR: 0.1138–0.1888) in the nonfailure group and 0.1902 (IQR: 0.1696–0.2564) in the failure group (*p* = 0.0003).

For the PL bundle, the median PLR was 0.0958 (IQR: 0.0706–0.1146) in the nonfailure group and 0.1367 (IQR: 0.1195–0.1484) in the failure group (*p* = 0.0004) (Table 2).

**Table 2:** Diagnostic performance of bundle morphology ratios for predicting graft failure.

Variable	AUC	95% CI	Cutoff	Sensitivity	Specificity
AMR	0.804	0.707–0.902	0.148	1.000	0.575
PLR	0.799	0.676–0.922	0.118	0.846	0.783

Abbreviations: AMR, anteromedial ratio; AUC, area under the curve; CI, confidence interval; PLR, posterolateral ratio.

### ROC curve analysis

**Figure 3:**
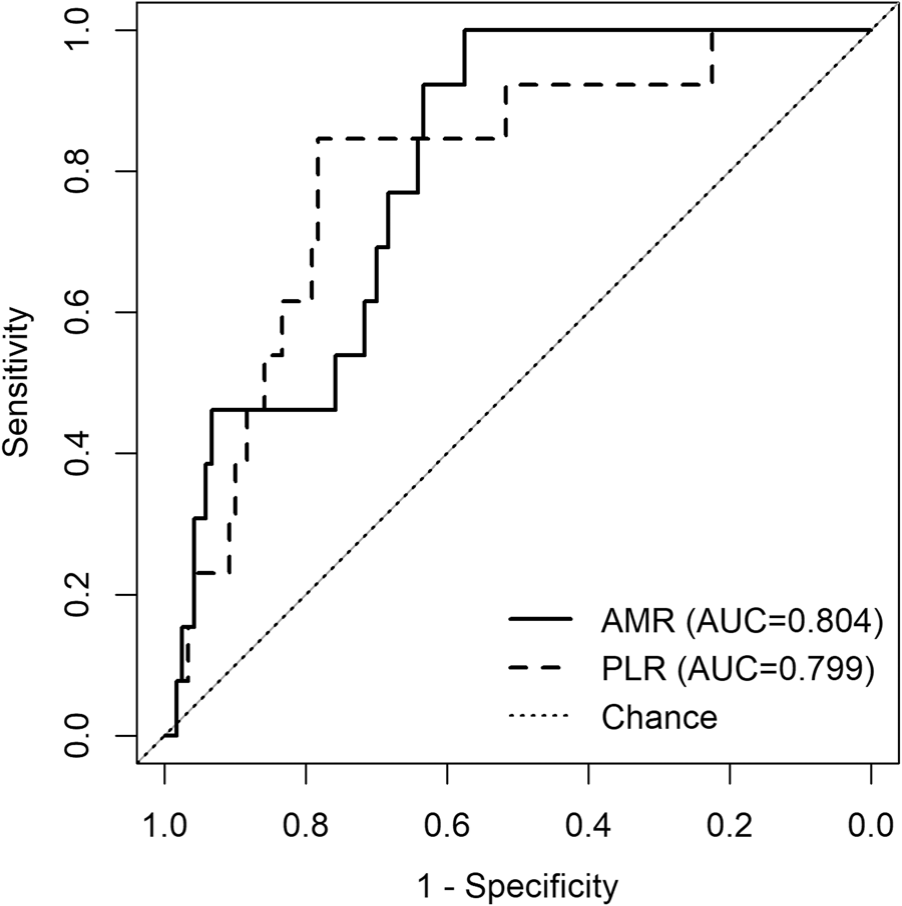
Receiver operating characteristic curves for predicting graft failure. ROC curves demonstrating the discriminative ability of anteromedial bundle ratio (AMR) and posterolateral bundle ratio (PLR) for predicting graft failure within 2 years. AUC values indicate moderate‐to‐good discrimination in this cohort. AUC, area under the curve; ROC, receiver operating characteristic.

ROC analysis demonstrated moderate‐to‐good discrimination in this cohort for both morphological parameters (Figure 3).

AMR showed an AUC of 0.804 (95% CI: 0.707–0.902), whereas PLR demonstrated an AUC of 0.799 (95% CI: 0.676–0.922). The optimal cutoff values determined by the Youden index were 0.148 for AMR (sensitivity 1.00, specificity 0.575) and 0.118 for PLR (sensitivity 0.846, specificity 0.783) (Table 2).

### Logistic regression analysis

Univariate logistic regression demonstrated significant associations between bundle morphology and graft failure (Table 3).

**Table 3:** Logistic regression analysis for graft failure. Univariable and multivariable logistic regression models evaluating associations between bundle morphology ratios and graft failure.

Variable	Univariable OR (95% CI)	*p*‐value	Multivariable OR (95% CI)	*p‐*value
AMR	1.21 (1.09–1.36)	<0.001	1.15 (1.02–1.31)	0.026
PLR	1.25 (1.09–1.44)	<0.001	1.12 (0.94–1.33)	0.189

Abbreviations: AMR, anteromedial ratio; CI, confidence interval; OR, odds ratio; PLR, posterolateral ratio.

Per 0.01‐unit increase in AMR, the odds of failure increased by 21% (OR: 1.21, 95% CI: 1.09–1.36); per 0.01‐unit increase in PLR, the odds increased by 25% (OR: 1.25, 95% CI: 1.09–1.44).

In multivariable logistic regression adjusting for age, sex, BMI, activity level and meniscal injury, AMR remained associated with graft failure in an exploratory multivariable model (*p* = 0.026), whereas PLR did not retain statistical significance.

The multivariable model demonstrated good discrimination (AUC = 0.831) and calibration (Hosmer–Lemeshow *p* = 0.81).

### Measurement reliability

**Figure 4:**
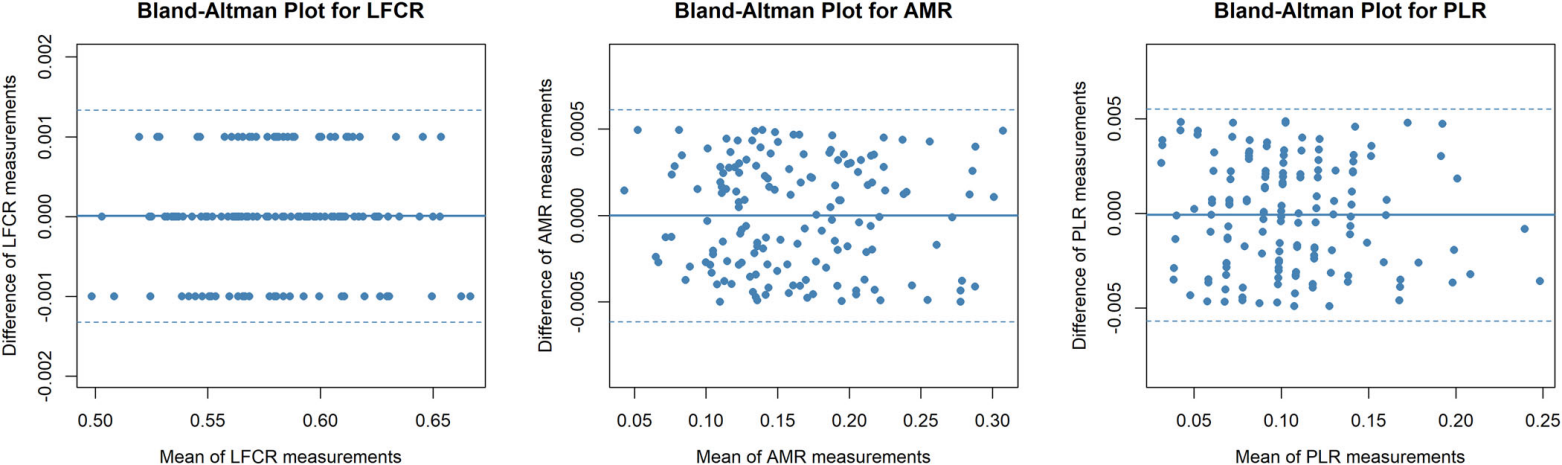
Bland–Altman analysis of intraobserver reliability. Bland–Altman plots showing agreement between repeated measurements of LFCR, AMR and PLR. The central solid line represents the mean difference, and dashed lines indicate the limits of agreement (±1.96 SD), demonstrating minimal systematic bias and no evidence of proportional bias across all parameters. AMR, anteromedial ratio; LFCR, lateral femoral condyle ratio; PLR, posterolateral ratio; SD, standard deviation.

Intraobserver reliability was good to excellent across all parameters. ICC values were 0.878 (95% CI: 0.835–0.910) for LFCR, 0.949 (95% CI: 0.930–0.963) for AMR and 0.922 (95% CI: 0.857–0.953) for PLR. Bland–Altman analysis demonstrated minimal systematic bias across all parameters, with mean differences close to zero and narrow limits of agreement. No significant proportional bias was observed for LFCR (*r* = −0.024, *p* = 0.77), AMR (*r* = 0.083, *p* = 0.31) or PLR (*r* = −0.041, *p* = 0.61), indicating that measurement variability was random rather than magnitude‐dependent (Figure 4).

## DISCUSSION

The most important finding of this study is that bundle‐specific morphology ratios derived from routine postoperative imaging were significantly associated with graft failure following anatomic double‐bundle ACL reconstruction. Both AMR and PLR demonstrated discriminative ability for predicting noncontact graft failure, indicating that tunnel positional morphology reflects biomechanically relevant conditions influencing graft integrity. These findings are consistent with previous studies highlighting the role of structural morphology in ACL injury and reinjury risk prediction [3, 4, 5, 6, 7, 8, 9], and extend these concepts to bundle‐specific postoperative positional indices.

Femoral morphology has been increasingly recognised as a structural risk factor for ACL injury. Pfeiffer et al. reported that an increased LFCR was associated with a higher risk of primary ACL injury, and Gao et al. [3, 9] further demonstrated that increased LFCR was linked to graft rerupture after reconstruction. Hodel et al. introduced the lateral femoral condyle index as an imaging‐based parameter for ACL injury risk, supporting the concept that subtle femoral morphological variations contribute to ligament vulnerability [5]. In addition, Jeon et al. suggested that LFCR may act synergistically with posterior tibial slope and notch morphology in determining ACL injury susceptibility [6]. It should be noted that anatomic double‐bundle reconstruction is performed with relatively high frequency at our institution, consistent with practice patterns in Japan, where this technique has been systematically refined over two decades [7]. While this approach is less commonly employed in Western centres, the morphological indices proposed here are derived from standard postoperative CT and are potentially applicable to any double‐bundle reconstruction technique regardless of geographic setting. The generalisability of these findings to single‐bundle reconstruction, however, warrants separate investigation.

The present study advances this line of research by extending morphology‐based risk assessment beyond native bony structure to postoperative graft configuration. Unlike previous investigations that focused primarily on primary injury risk, we specifically evaluated bundle‐specific tunnel morphology and its association with noncontact graft failure. By restricting the failure group to noncontact mechanisms, we aimed to better isolate intrinsic structural and biomechanical risk factors while minimising confounding by direct traumatic events.

Our ROC analysis demonstrated moderate‐to‐good discrimination, comparable to previously reported structural risk indicators. Importantly, measurement reproducibility was excellent, indicating that these indices can be reliably obtained from standard imaging without additional protocols. This supports the practical applicability of quantitative morphology assessment in routine clinical settings.

Multivariable analysis revealed that although both AMR and PLR were associated with failure in univariate models, only AMR retained the strongest association with graft failure after adjustment for confounders in the exploratory multivariable model. This finding suggests that AM bundle positioning may play a more dominant biomechanical role in postoperative graft stability than the PL bundle.

The median time to failure of 10 months (IQR: 8–12 months) coincides with the typical return‐to‐sport period following ACL reconstruction, suggesting that dynamic loading during athletic activity may be a contributing factor to graft failure in this population. From a clinical perspective, identification of morphology patterns associated with increased failure risk may aid surgeons in evaluating femoral tunnel positioning and reconstruction quality. If AMR is found to be elevated on routine postoperative CT, surgeons may consider this information when counselling patients about reinjury risk and planning rehabilitation or return‐to‐sport protocols. Whether intraoperative modification of tunnel position could mitigate this risk remains to be investigated in prospective studies. It should also be noted that routine postoperative CT involves radiation exposure, which carries particular relevance in adolescent populations. Future work should explore whether MRI‐based measurement protocols could provide equivalent morphological information with reduced radiation burden. While causality cannot be established in this retrospective design, the observed associations provide a rationale for prospective validation and potential integration with functional or biomechanical assessments.

Several limitations warrant consideration. First, this was a single‐centre retrospective study, which may limit external validity, and the findings may not generalise to centres where double‐bundle reconstruction is infrequently performed. Second, the limited number of failure events (*n* = 13) restricts statistical power for multivariable modelling and raises concern for potential overfitting; no external validation was performed. Third, interobserver reliability was not assessed, which may limit the reproducibility of the proposed indices across different operators. Fourth, the exclusion of patients with LFCR ≥ 62.18% may introduce selection bias by creating an artificially lower‐risk cohort and limiting external validity. Fifth, the classification of contact versus noncontact reinjury mechanism relied on medical records and patient‐reported history, which may be subject to misclassification. Sixth, data on return‐to‐sport timing and sports exposure were not systematically collected, limiting the assessment of activity‐related confounders. Seventh, contralateral ACL injury was not analysed as a covariate. Eighth, potential confounding from surgical learning curve or contributions from multiple surgeons was not formally assessed. Ninth, routine postoperative CT involves radiation exposure, which warrants consideration in adolescent patients; the risk‐benefit profile of CT in this context should be evaluated in future prospective studies. All meniscal injuries identified at the time of surgery were treated with repair; no meniscectomies were performed in this cohort.

Despite these limitations, the study includes a consecutive cohort, standardised imaging methodology based on established protocols, and robust reliability analysis. These strengths support the validity of the findings and highlight the potential of imaging‐derived bundle morphology as a clinically accessible parameter for postoperative risk stratification.

## CONCLUSION

Postoperative CT‐derived bundle morphology ratios are associated with noncontact graft failure following anatomic double‐bundle ACL reconstruction and demonstrate good‐to‐excellent reproducibility. These preliminary findings suggest that quantitative morphology ratios may be useful for postoperative risk stratification, but prospective validation in larger, multicenter cohorts is required before clinical implementation.

## AUTHOR CONTRIBUTIONS

Yuko Takeuchi conceived the study, performed data collection and analysis and drafted the manuscript. Ryosuke Kawai, Ryosuke Kawai and Takashi Tsukahara contributed to surgical procedures, data interpretation and critical revision of the manuscript. All authors approved the final version.

## FUNDING INFORMATION

The authors have no funding to report.

## CONFLICT OF INTEREST STATEMENT

The authors declare no conflict of interest.

## ETHICS STATEMENT

This study was conducted in accordance with the Declaration of Helsinki and was approved by the Ethics Committee of Asahi University Hospital (Research No. 2026‐01‐01). The requirement for individual informed consent was waived due to the retrospective nature of the study. This study was conducted in accordance with the Declaration of Helsinki and was approved by the Ethics Committee of Asahi University Hospital (Research No. 2026‐01‐01). The requirement for individual informed consent was waived due to the retrospective nature of the study. The requirement for individual informed consent was waived by the Ethics Committee of Asahi University Hospital due to the retrospective nature of the study.

## Data Availability

Data are available from the corresponding author upon reasonable request.
